# Supplementary material for: Venomix: a simple bioinformatic pipeline for identifying and characterizing toxin gene candidates from transcriptomic data
Source: PeerJ. 2018 Jul 31;6:e5361. doi: 10.7717/peerj.5361 (PMC6074769; doi:10.7717/peerj.5361)
Supplement: Supplemental Information 3 [file peerj-06-5361-s003.gz › FinalOutput_E-20/C-type_lectin_galatrox_1/finaltree.pdf]

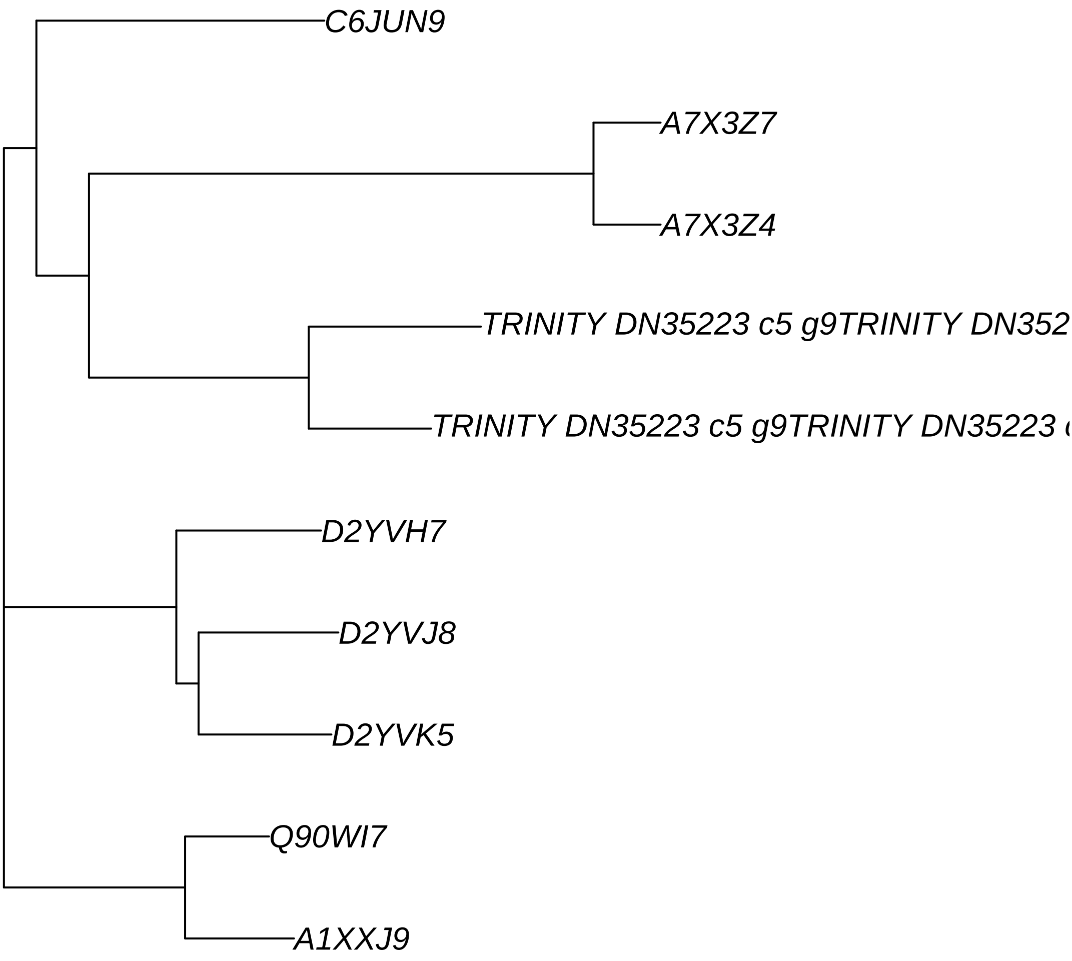

C6JUN9
A7X3Z7
A7X3Z4
TRINITY DN35223 c5 g9TRINITY DN352
TRINITY DN35223 c5 g9TRINITY DN35223
D2YVH7
D2YVJ8
D2YVK5
Q90WI7
A1XXJ9
